# Axonal degeneration in an Alzheimer mouse model is PS1 gene dose dependent and linked to intraneuronal Aβ accumulation

**DOI:** 10.3389/fnagi.2014.00139

**Published:** 2014-06-27

**Authors:** Ditte Z. Christensen, Melanie Huettenrauch, Miso Mitkovski, Laurent Pradier, Oliver Wirths

**Affiliations:** ^1^Division of Molecular Psychiatry, Department of Psychiatry and Psychotherapy, University Medical Center (UMG), Georg-August-UniversityGoettingen, Germany; ^2^Light Microscopy Facility, Max-Planck-Institute of Experimental MedicineGoettingen, Germany; ^3^Central Nervous System Department, Centre de Recherche Vitry-Alfortville, Sanofi-AventisVitry-sur-Seine, France

**Keywords:** amyloid, presenilin, axonal degeneration, axonopathy, intraneuronal Abeta, transgenic mice, Alzheimer, axonal transport

## Abstract

Abnormalities and impairments in axonal transport are suggested to strongly contribute to the pathological alterations underlying AD. The exact mechanisms leading to axonopathy are currently unclear, but it was recently suggested that APP expression itself triggers axonal degeneration. We used APP transgenic mice and crossed them on a hemi- or homozygous PS1 knock-in background (APP/PS1KI). Depending on the mutant PS1 dosage, we demonstrate a clear aggravation in both plaque-associated and plaque-distant axonal degeneration, despite of an unchanged APP expression level. Amyloid-β (Aβ) peptides were found to accumulate in axonal swellings as well as in axons and apical dendrites proximate to neurons accumulating intraneuronal Aβ in their cell bodies. This suggests that Aβ can be transported within neurites thereby contributing to axonal deficits. In addition, diffuse extracellular Aβ deposits were observed in the close vicinity of axonal spheroids accumulating intracellular Aβ, which might be indicative of a local Aβ release from sites of axonal damage.

## Introduction

Alzheimer's disease (AD) is a severe neurodegenerative disorder characterized by progressive cognitive decline representing the main cause of dementia among the elderly population. Major neuropathological hallmarks of AD include the deposition of extracellular senile plaques, consisting of amyloid-β (Aβ) peptides and the occurrence of neurofibrillary tangles, representing intracellular accumulations of abnormal phosphorylated protein tau (Selkoe, 2001). While the majority of all AD cases occurs sporadically, a small percentage of familial early onset cases is caused by mutations in either the amyloid-precursor protein (APP) or the presenilin 1 (PS1) or presenilin 2 (PS2) genes. Most of these mutations have been shown to elevate the levels of the pathologically relevant Aβ 42 isoform, which is considered to cause neurotoxicity and shows a tendency to aggregate very rapidly into amyloid fibrils (Iwatsubo, 1998; St. George-Hyslop, 2000).

Impairments in axonal transport with the formation of axonal spheroids have been reported in a variety of neurodegenerative diseases (Yagishita, 1978) and are suggested to strongly contribute to the pathological alterations underlying AD (Roy et al., 2005; Zhu et al., 2005; Stokin and Goldstein, 2006) and other diseases like e.g., lysosomal storage disorders (Ohara et al., 2004). Fundamental axonal pathology in AD includes the aberrant accumulation of various proteins in abnormal swollen axons, including APP (Cras et al., 1991), synaptic proteins like alpha-synuclein (Wirths et al., 2000), glycogen (Mann et al., 1987), as well as the occurrence of abnormal paired helical filaments (PHFs) (Praprotnik et al., 1996). With the implementation of new imaging techniques, it has been recently shown in *in-vivo* studies that impairment of axonal transport mechanisms and decreased axonal transport rates might have a significant impact on the pathogenesis of AD already early in the disease process (Smith et al., 2003; Teipel et al., 2007; Cross et al., 2008; Minoshima and Cross, 2008).

Indications for disturbances in axonal transport with concomitant axonopathy have been described in different APP-based transgenic AD mouse models (Stokin et al., 2005; Salehi et al., 2006; Wirths et al., 2006, 2007; Adalbert et al., 2009; Chen et al., 2011; Jawhar et al., 2012). Surprisingly, it has been reported that an increase in the Aβ 42/Aβ 40 ratio, as well as increased deposition of Aβ peptides, resulted in a suppression of APP-induced axonal deficits in transgenic mouse and *Drosophila* models, leading to the suggestion that axonal defects are not caused by Aβ peptides but depend entirely on APP expression levels (Stokin et al., 2008). To investigate this further, we quantified axonal spheroids in APP single transgenic and APP transgenic mice co-expressing knocked-in mutant PS1 on endogenous levels in either a hemi- (APP/PS1KI^he^) or homozygous (APP/PS1KI^ho^) manner in the present report. Aβ peptide levels were dramatically increased as a function of PS1 knock-in gene dosage and led to a significant aggravation of the axonal phenotype, despite of unchanged APP expression levels. In addition, we provide evidence for a functional relationship between intraneuronal accumulation of Aβ peptides and the formation of plaque-distant axonal spheroids by means of confocal microscopy in APP/PS1KI^he^/YFP-H transgenic mice.

## Materials and methods

### Transgenic mice

The generation of APP/PS1KI mice has been described previously (Casas et al., 2004). In brief, human mutant APP751 containing the Swedish and London mutations is overexpressed under the control of the murine Thy-1 promoter, whereas murine PS1 with the M233T and L235P FAD-linked mutations is expressed under the control of the endogenous mouse PS1 promoter. Mice designated APP were hemizygous for the APP751SL transgene, whereas in APP/PS1KI^he^ and APP/PS1KI^ho^ mice additionally one or both endogenous wildtype PS1 alleles were replaced by murine PS1 carrying the M233T and L235P mutations by a knock-in strategy. Mice designated PS1KI^ho^ were homozygous for the PS1 knock-in mutations without overexpression of human APP. All mice were used at the age of 10 months. To obtain APP/PS1KI^he^/YFP-H transgenic mice, APP/PS1KI^ho^ mice were crossed with homozygous YFP-H mice [line B6.Cg-Tg(Thy1-YFPH)2Jrs/J, Charles River Laboratories], expressing the fluorescent protein YFP in a subset of neurons (Feng et al., 2000). All animals were handled according to German guidelines for animal care.

### Immunohistochemistry on paraffin sections

Mice were transcardially perfused with 4% paraformaldehyde (PFA) in 0.01 M phosphate buffered saline (PBS) and brains and spinal cords were carefully dissected. Post fixation was carried out in 4% buffered formalin at 4°C before the tissue was embedded in paraffin. Immunohistochemistry was performed on 4 μm paraffin sections, as described previously (Wirths et al., 2002). In brief, sections were deparaffinized in xylene and rehydrated in an ethanol series. After treatment with 0.3% H_2_O_2_ in PBS to block endogenous peroxidases, antigen retrieval was achieved by boiling sections in 0.01 M citrate buffer pH 6.0, followed by 3 min incubation in 88% formic acid. Non-specific binding sites were blocked by treatment with skim milk and fetal calf serum in PBS prior to the addition of the primary antibodies. The following antibodies were applied: 4G8 (Covance, 1:10.000) (Christensen et al., 2008), OC (generous gift of Glabe and Kayed, 1:1000) (Kayed et al., 2007) and Aβ [N] (IBL, 1:500) (Christensen et al., 2010) against Aβ, 22C11 (Millipore, 1:1000) and 23850 (generous gift of G. Multhaup, 1:500) against human APP, G2-10 (Millipore, 1:500) and Aβ 40 (Synaptic Systems, #218203, 1:500) against Aβ 40, Aβ 42 (Synaptic Systems, #218703, 1:250), N3pE against pyroglutamate-modified Aβ (American Research Products, 1:250) (Wirths et al., 2009), kinesin light chain 1 (KLC1, Santa Cruz, sc-25735, 1:100) as well as anti-NF-200 (Millipore, 1:1000) and an antibody detecting phosphorylated APP at position T668 (anti-pT668; Cell Signaling Technologies, 1:500). Primary antibodies were incubated overnight in a humid chamber at room temperature followed by incubation with biotinylated secondary antibodies (DAKO). Staining was visualized using the ABC method with Vectastain kit (Vector Laboratories) and diaminobenzidine (DAB) as chromogen providing a reddish brown color. For double staining, the procedure was repeated starting from blocking with skim milk followed by incubation with the second primary antibody and ended by combining the ABC Vectastain kit with Histogreen (Linaris) instead of DAB, providing a green color. Counterstaining was carried out with hematoxylin. PS1KI^ho^ control mice were consistently negative for APP and Aβ staining.

### Western-blot

For immunoblotting, proteins were extracted in lysis buffer (50 mM Tris, 120 mM NaCl, pH 8.0) and electrophoresis was performed using 4–12% sodium dodecylsulfate-polyacrylamide Variogels (Anamed) under denaturing conditions. Proteins were transferred to nitrocellulose membranes (Amersham Pharmacia) which were blocked with 10% skim milk in Tris buffer and blots were probed with the monoclonal Aβ antibody W0-2 (Millipore), detecting full-length APP, C99, and Aβ peptides.

### Quantification of Aβ load

For each animal, three paraffin embedded sections at least 25 μm afar from each other were stained simultaneously with DAB as chromogen using the 4G8 antibody (1:10.000). Quantification of the Aβ load was performed using an Olympus BX-51 microscope equipped with an Olympus DP-50 camera and the ImageJ software [NIH, USA (Wayne Rasband. ImageJ. U.S. National Institutes of Health, Bethesda, Maryland, USA, http://rsb.info.nih.gov/ij/, 1997–2013)], and was performed for 6 mice each of the genotypes APP, APP/PS1KI^he^, and APP/PS1^ho^. The area fraction covered by Aβ staining was evaluated in the frontal cortex providing a general measure of the Aβ accumulation. Furthermore, the area covered by Aβ staining was evaluated in pons and spinal cord where axonal pathology was quantified. Representative 100x magnified images were systematically captured in each region. The area fraction calculation was based on binarized images generated with ImageJ through a fixed intensity threshold selective for the 4G8 DAB stain.

### Quantification of plaque-distant axonal swellings

For each animal, two series of three paraffin embedded sections at least 25 μm afar were stained simultaneously using either the NF-200 or the anti-pT668 antibodies with DAB as chromogen together with the 4G8 antibody using Histogreen as chromogen. Quantification of plaque-distant dystrophic fibers with a diameter of >10 μm was performed using the meander scan of StereoInvestigator 7 (MicroBrightfield) and a BX51 microscope (Olympus). On average, three sections were used for statistical analyses. Quantifications were performed in pons and spinal cord because these regions had obvious dystrophic fiber pathology together with moderate plaque pathology, making it possible to distinguish plaque associated from plaque independent dystrophic neurites. Quantification was performed in 6 mice of each of the genotypes APP, APP/PS1KI^he^ and APP/PS1KI^ho^ thus having the same levels of the APP transgene but having varying PS1KI gene dosages.

### Confocal microscopy

APP/PS1KI^he^/YFP-H mice were stained using the OC antibody detecting Aβ fibrillar oligomers (Kayed et al., 2007). Images were acquired with an AOBS-equipped Leica SP2 confocal laser scanning microscope (Leica Microsystems, Mannheim, Germany) using a 63×/1.4 oil-immersion objective. Sequential, line-by-line 405, 514, and 561 nm laser scans were applied to collect the respective DAPI (410–480 nm), YFP (520–550 nm), and Alexa Fluor 568 (580–650 nm) emissions. The ImageJ software package was then used to view and process the resulting images with a noise-reducing, median filter.

### Real time-PCR

For real-time RT-PCR analysis, 10-month-old male mice (*n* = 4 per group) were used. In brief, frozen brain hemispheres were homogenized in 1 ml of TriFast reagent (Peqlab) per 100 mg tissue using a glass-teflon homogenizer (10 strokes, 800 rpm). RNA extraction and DNAse digestion was performed according to the protocol of the manufacturer. Reverse transcription of the purified RNA samples was carried out using the First Strand cDNA Synthesis Kit (ThermoFisher). RT-PCR was performed using a Stratagene MX3000P Real-Time Cycler. The SYBR-green based *FastStart Universal* SYBR Green (Roche) containing ROX as an internal reference dye was used for amplification. The following primer sets were used: KLC1-for: CTTCCTCCCCTCCGTCAG, KLC1-rev: AAATGACCCTGAGAGCATGG; KIF1A-for: ACCGCCACCTTCACAGAG, KIF1A-rev: CTATCCCCAGTGACGCCTG; KIF5A-for: GGACGTCTTTGACGATCTGC, KIF5A-rev: GGGACGACAGCGTCATTATT. Relative expression levels were calculated using the 2^−ΔΔ Ct^ method (Livak and Schmittgen, 2001). Expression levels were normalized to the housekeeping gene β-Actin and calibrated to average expression level of untreated wild type animals for each gene (Hillmann et al., 2012).

### Statistical analysis

Dystrophic fiber quantifications and Aβ area percentages were analyzed using One-Way ANOVA followed by *t*-tests. Data are presented as mean ±s.e.m. Significance levels were given as follows: ^***^P < 0.001; ^**^P < 0.01; ^*^P < 0.05. All statistical analysis was performed using GraphPad Prism version 4.03 for Windows (GraphPad Software, San Diego, CA, USA).

## Results

### Analysis of APP expression levels and Aβ load with increasing mutant PS1 gene dosage

**Figure 1:**
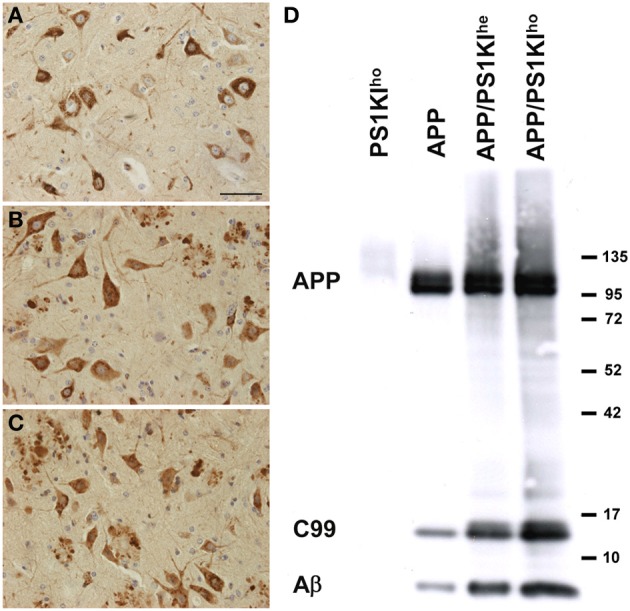
**Immunostaining of human APP using 22C11 in spinal cords of APP (A), APP/PS1KI^he^**(B)**, and APP/PS1KI^ho^**(C)** mice showing comparable numbers of APP-expressing cells in all three genotypes**. Western-blot analyses using W0-2 revealed no differences in the amount of human full-length APP among the three genotypes, however, the levels of C99 and Aβ increased with increasing PS1KI gene dosage **(D)**. Scale bar: 50 μm.

Staining with antibodies against human APP revealed approximately equal numbers of APP-positive cells in APP, APP/PS1KI^he^, and APP/PS1KI^ho^ mice at 10 months of age with comparable staining intensity, but with dystrophic fibers accumulating APP in APP/PS1KI^he^ and APP/PS1KI^ho^ mice (Figures 1A–C). Western-blot analysis confirmed that the overall expression of human full-length APP in the three mouse lines was comparable with no considerable variation between the different genotypes. In contrast, the levels of the β-secretase cleavage product C99, as well as Aβ, increased with incremental mutant PS1 gene dosage (Figure 1D).

**Figure 2:**
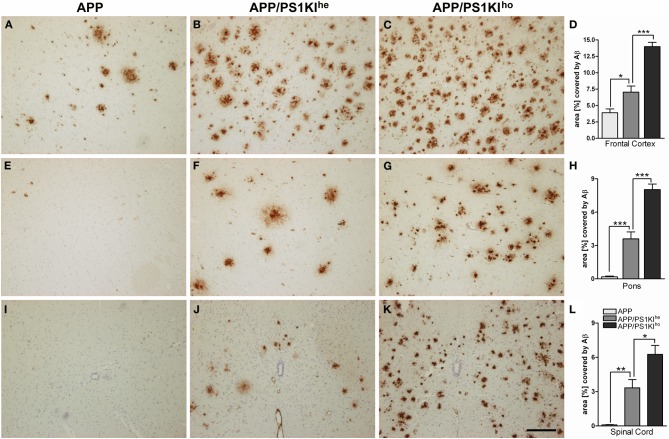
**Immunostaining of Aβ using 4G8 antibody in 10-month-old APP, APP/PS1KI^he^, and APP/PS1KI^ho^ mice in frontal cortex (A–C), pons (E–G), and spinal cord (I–K) together with respective quantifications of the area covered by Aβ staining (D,H,L)**. Virtually no Aβ peptide accumulation was detected in the pons and spinal cord of APP mice, whereas about 4% of the frontal cortical area was covered by Aβ staining. Upon introduction of one PS1KI gene (APP/PS1KI^he^ mice), the accumulation of Aβ was found to rise significantly in all three regions investigated and further approximately doubled with the introduction of an additional PS1KI gene (APP/PS1KI^ho^ mice), reaching percentile Aβ covered areas of 14, 8, and 6.5% in the frontal cortex, pons, and spinal cord, respectively. Data was analyzed from 6 mice of each genotype and analyzed using One-Way ANOVA followed by *t*-tests. All error bars represent mean ±s.e.m. ^***^P < 0.001; ^**^P < 0.01; ^*^P < 0.05. Scale bar: 200 μm.

To assess the influence of varying gene doses of PS1 on Aβ levels, Aβ plaque pathology was quantified in the frontal cortex, pons and spinal cords of 10-month-old APP, APP/PS1KI^he^, and APP/PS1KI^ho^ mice using computer-assisted analysis of 4G8 DAB stained sections (Figure 2). Only very minor amounts of Aβ were observed in the pons and spinal cords of APP transgenic mice harboring no PS1KI mutations (Figures 2E,H,I,L), whereas 3.9% of the frontal cortex were covered by Aβ (Figures 2A,D). Introducing one PS1KI allele to generate APP/PS1KI^he^ mice resulted in a significant increase in the Aβ load in the frontal cortex by 80% leading to an overall Aβ load of 7% (Figure 2A). In good agreement, significant Aβ amounts were observed in the pons and spinal cord which had Aβ loads of 3.6 and 3.3%, respectively (Figures 2F,H,J,L). A further increase in the PS1KI gene dosage resulting in the APP/PS1KI^ho^ mice caused another approximate doubling of the Aβ load in all three regions measured (Figures 2C,D,G,H,K,L), whereas PS1KI^ho^ mice were consistently negative for 4G8 staining (data not shown). The introduction of one or two copies of mutant PS1 under the control of its endogenous promoter therefore strongly aggravates extracellular Aβ pathology.

### Phosphorylated APP (pAPP) as a marker for dystrophic neurites and axonal spheroids

**Figure 3:**
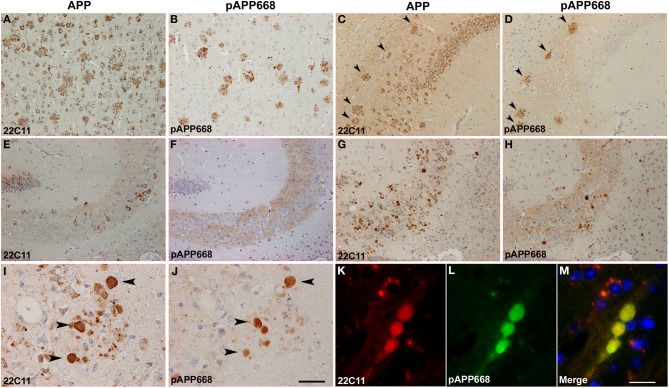
**Comparison between APP (22C11) and phosphorylated APP (pAPP668) staining patterns using parallel sections in different brain regions of APP/PS1KI^ho^ mice of different ages**. Whereas 22C11 stains dystrophic neurites as well as cell bodies, pAPP668 exclusively stains axonal compartments in cortex (**A,B**: 2 m), CA1 (**C,D**: 2 m), hippocampal mossy fibers (**E,F**: 2 m; **G,H**: 10 m; **I,J**: 14 m). Double-immunofluorescent staining of 22C11 **(K)** and pAPP668 **(L)** shows a high co-localization in axonal swellings **(M)**. Arrowheads indicate same plaques **(C,D)** or axonal swellings **(I,J)**. Scale bars: **(A–H)**: 100 μm; **(I,J)**: 33 μm; **(K–M)**: 20 μm.

APP represents a widely used marker for the detection of axonal pathology e.g., in the analysis of traumatic brain injury (TBI) (Pierce et al., 1996), which has also been previously used to detect axonal degeneration in APP/PS1KI^ho^ mice (Wirths et al., 2007). However, one of the problems of using APP antibodies in the analysis of axonal degeneration in APP-transgenic mouse models is the very broad staining pattern. This includes cell bodies, axonal swellings, and dystrophic neurites in the vicinity of amyloid plaques, thereby complicating the analysis of distinct pathological alterations like axonal pathology especially in plaque-rich brain areas. By using an antibody which detects phosphorylated APP T668 (anti-pT668), no staining of cell somata, but only plaque-associated dystrophic neurites, as well as large plaque-distant axonal swellings could be detected. Staining of adjacent sections with antibodies detecting an N-terminal epitope of APP (Figures 3A,C,E,G,I,K) and anti-pT668 (Figures 3B,D,F,H,J,L) revealed a complete overlap of plaque-associated neurites and isolated axonal spheroids. APP phosphorylated at position 668 is localized mainly in axonal processes, as is evident in the mossy fibers of the hippocampus (Figures 3F,H), in contrast to APP which is mainly found in the somatodendritic compartment (Figures 3E,G). Double-immunofluorescent staining revealed a nice co-localization in axonal spheroids (Figures 3K–M). The use of pAPP antibodies strongly facilitates the quantification of axonal spheroids as it represents an excellent marker labeling only axonal processes without concomitant staining of cell bodies.

### Quantification of dystrophic fibers with varying PS1KI gene dosage

**Figure 4:**
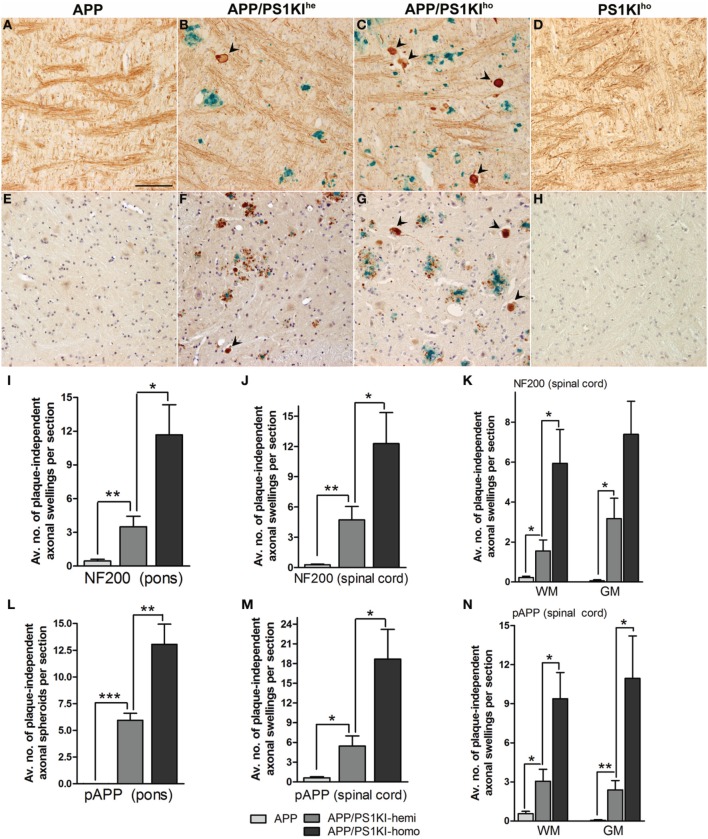
**Double ABC-immunostaining showing Aβ in blue using 4G8 antibodies together with visualization of fibers in reddish brown using either NF-200 or anti-pT668**. The number of plaque-distant dystrophic neurites (black arrowheads) was found to increase with PS1KI gene dosage; examples of stainings from APP, APP/PS1KI^he^, APP/PS1KI^ho^, as well as PS1KI^ho^ mice are shown using the NF-200 antibody in the pons **(A–D)** and anti-pT668 antibody in the spinal cord **(E–H)**. Increasing numbers of NF-200 **(I–K)** as well as pT668-positive **(L–N)** plaque-distant dystrophic fibers were found with increasing PS1 gene dosage in both pons and spinal cord with practically no dystrophic fibers in the APP mice. All error bars represent mean ±s.e.m. ^***^P < 0.001; ^**^P < 0.01; ^*^P < 0.05. Scale bar: 100 μm.

The number of plaque-distant axonal swellings with a diameter of larger than 10 μm was quantified in the pons and spinal cord using antibodies towards pAPP (anti-pT668) and neurofilament (NF-200) as markers of axonopathy. This was done in double-stainings together with 4G8 visualizing Aβ plaques in 10-month-old APP, APP/PS1KI^he^, and APP/PS1KI^ho^ mice in the pons (Figures 4A–C) and spinal cord (Figures 4E–G), to ensure that only plaque-distant axonal spheroids were counted. The quantification showed practically no axonal swellings in the pons and spinal cord of APP single transgenic mice with either NF-200 (Figures 4I–K) or anti-pT668 (Figures 4L–N). In contrast, a considerable amount of dystrophic fibers was found in the APP/PS1KI^he^ mice using both NF-200 and anti-pT668 in pons as well as spinal cord, where the increase was found in both gray and white matter, leading to an even further increase in APP/PS1KI^ho^ mice (Figure 4). No dystrophic fibers were found in PS1KI^ho^ (Figures 4D,H) or wildtype control mice (not shown). Thus using the two markers NF-200 and anti-pT668, the amount of dystrophic fibers was found to rise proportionally to the dosage of the mutant PS1KI gene in APP transgenic mice, however, this type of pathological alterations is completely lacking in mice harboring the PS1KI^ho^ gene alone.

### Linkage of intraneuronal and intraaxonal accumulation of Aβ peptides

**Figure 5:**
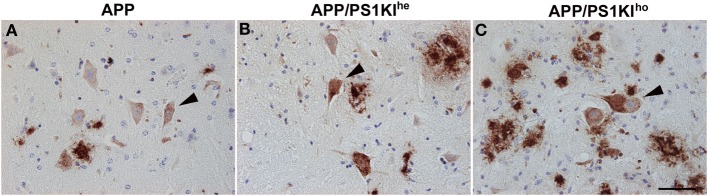
**Intraneuronal Aβ accumulation (detected by 4G8, arrowheads) in the spinal cord was found to rise proportionally with increasing PS1KI gene dosage in 10-month-old APP (A), APP/PS1KI^he^ (B), and APP/PS1KI^ho^ mice (C)**. Scale bar: 50 μm.

**Figure 6:**
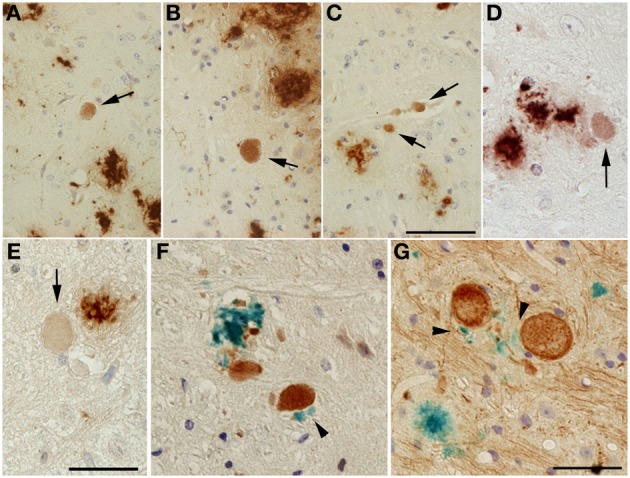
**Aβ-positive axonal swellings (arrows) (A–C)**. Axonal swellings were stained with 4G8 **(A,B)**, Aβ [N] **(C)** or end-specific antibodies detecting Aβ 40 **(D)** or Aβ N3pE **(E)**. Double immunostaining of Aβ (4G8, blue) together with either anti-pT668 **(F)** or NF-200 **(G)** (reddish brown). Small Aβ deposits (black arrowheads) could occasionally be detected in the vicinity of large dystrophic neurites in both APP/PS1KI^he^ and APP/PS1KI^ho^ mice. Scale bar: **(A–C)**: 50 μm; **(D,E)**: 33 μm; **(F,G)**: 12.5 μm.

**Figure 7:**
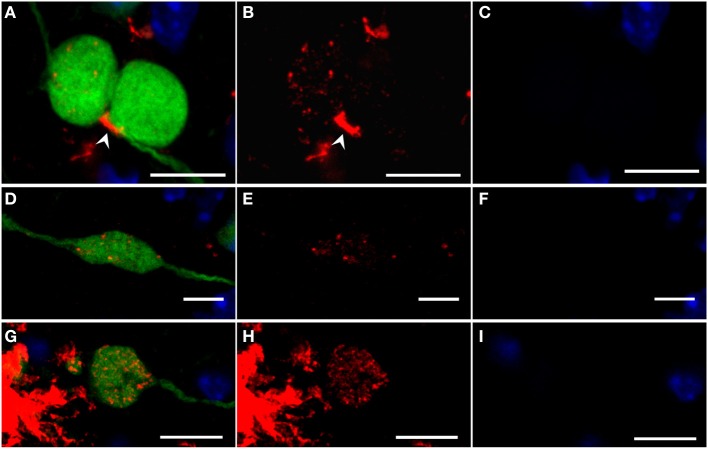
**Confocal images of fibrillar Aβ oligomers and Aβ fibrils (OC antibody, red) accumulating in large dystrophic fibers (YFP, green) of 6-month-old YFP/APP/PS1KI^he^ mice with DAPI counterstaining confirming the lack of nuclei (blue)**. Merged images are shown **(A,D,G)** together with the isolated OC **(B,E,H)**, and DAPI staining **(C,F, I)**. Extracellular accumulation of Aβ (White arrowhead) could be detected near large dystrophic fibers accumulating intracellular Aβ **(A–C)**. The intracellular accumulation of intracellular granules of OC-stained Aβ peptides were observed in many large dystrophic neurites, both plaque-distant **(D–F)** as well as occasionally in the vicinity of plaques **(G–I)**. **(A–C)** are generated as a maximum projection of 20 confocal images within the dystrophic fibers, whereas **(D–I)** are images of a single confocal plane. Scale bars: 10 μm.

The directly proportional rise in Aβ load observed with increasing PS1KI gene dosage in mice hemizygous for the APP transgene also relates to intraneuronal Aβ, as exemplified in motor neurons of the spinal cord (Figures 5A–C). In addition, axonal spheroids in APP/PS1KI^he^ and APP/PS1KI^ho^ mice stained positive with either 4G8 (Figures 6A,B), Aβ [N] (Figure 6C) or end-specific antibodies detecting neo-epitopes against Aβ 40 at the C-terminus (Figure 6D) and Aβ N3pE at the N-terminus (Figure 6E) of Aβ, while we were unable to detect Aβ 42-positive axonal spheroids, which might be due to a lower sensitivity of the used antibody in stainings using paraffin-embedded material (data not shown). To assess whether APP C-terminal fragments (CTFs) were present in axonal spheroids, we performed double-stainings using N- and C-terminal APP-specific antibodies. We detected a complete overlap, making it difficult to draw conclusions about the presence of APP C-terminal fragments only, as full-length APP is highly abundant in axons (data not shown). Using a combination of anti-pT668 and 4G8 (Figure 6F) or NF-200 and 4G8 (Figure 6G) in a conventional double-labeling, Aβ could be detected in the close vicinity of plaque-distant axonal spheroids (arrowheads), which might indicate that axonal swellings are able to release Aβ peptides locally. Such a mechanism would be supported by the analysis of APP/PS1KI^he^/YFP-H mice by confocal imaging. Using the OC antibody recognizing fibrillar Aβ oligomers, an accumulation of Aβ inside and in the close vicinity of large plaque-distant axonal spheroids was detected, although the axonal origin of the extracellular Aβ cannot be definitely proven by the applied methodology (Figures 7A–C).

**Figure 8:**
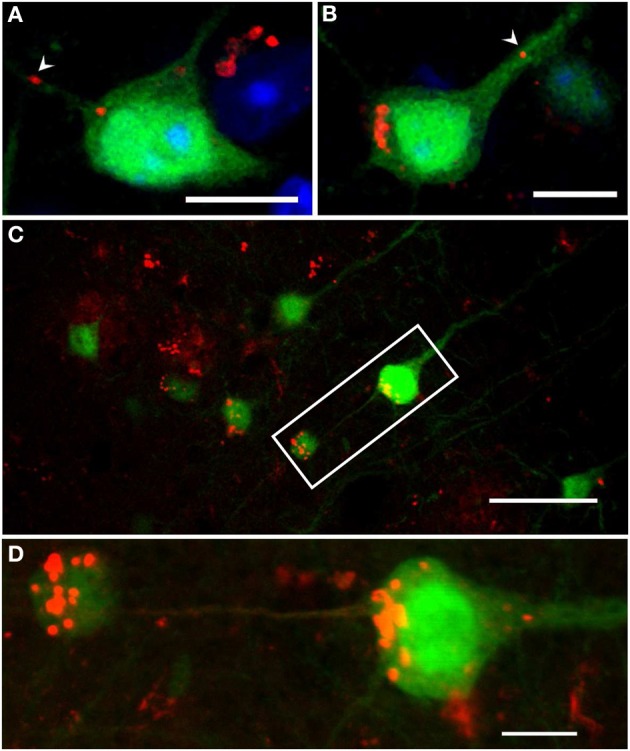
**Confocal images of intraneuronal Aβ accumulation in cortical neurons of 6-month-old YFP/APP/PS1KI^he^ mice**. Aβ accumulated inside cell bodies at the axon hillock **(A,B)**, as well as in axons (**A**, arrowhead) and large apical dendrites (**B**, arrowhead) projecting from the cell body (OC antibody, red). Also, axonal swellings accumulating intracellular Aβ were found directly connected to cortical neurons accumulating abundant amounts of intraneuronal Aβ at the axon hillock (Aβ [N] antibody, red) (**C,D**: enlargement of **C**). Scale bars: **(A,B,D)**: 10 μm; **(C)**: 40 μm.

**Figure 9:**
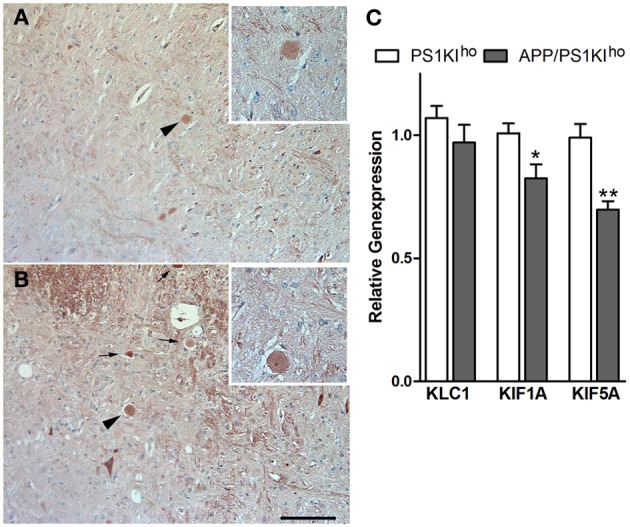
**KLC1-positive axonal spheroids (arrows) in 10-month-old APP/PS1KI^he^ (A) and APP/PS1KI^ho^ mice (higher magnification of spheroid indicated by arrowhead in inset)**. RT-PCR analysis of brain mRNA levels of kinesin family members revealed significantly reduced expression of KIF1A (*p* < 0.05) and KIF5A (*p* < 0.01) in APP/PS1Ki^ho^ mice compared to PS1KI^ho^ control mice **(C)**. All error bars represent mean ±s.e.m. ^**^P < 0.01; ^*^P < 0.05. Scale bar: **(A,B)**:100 μm

In general, many plaque-distant swollen axonal processes were found to accumulate Aβ, showing a scattered staining of Aβ-positive granules throughout the lumen (Figures 7D–F). The same pattern could also be detected in large dystrophic neurites in the vicinity of plaques (Figures 7G–I). Abundant accumulation of oligomeric intraneuronal Aβ inside cell bodies, especially in the cortex, was confirmed using the OC antibody, where Aβ granules were also observed in the axon (Figure 8A) and large apical dendrite (Figure 8B) projecting from cells, indicating that Aβ can be transported into axons as well as dendrites. Occasionally, axonal swellings accumulating oligomeric Aβ could be found directly connected to cortical neurons accumulating abundant amounts of intraneuronal Aβ at the axon hillock (Figures 8C,D). This might indicate that the Aβ accumulating in large plaque-distant axonal spheroids originates from neuronal cell bodies from where it becomes axonally transported. We additionally performed stainings of kinesin light chain 1 (KLC1) which has been previously linked to axonal transport of APP (Kamal et al., 2000). KLC1-positive axonal spheroids were detected in APP/PS1KI^he^ and APP/PS1KI^ho^ mice, being most abundant in the latter genotype (Figures 9A,B). We further analyzed brain mRNA expression levels of several components of the axonal transport machinery including KLC1 and kinesin family members KIF1A and KIF5A. Whereas no significant differences were detected in KLC1 mRNA levels, both KIF1A (*P* < 0.05) and KIF5A levels (*P* < 0.01) were reduced in APP/PS1KI^ho^ mice in comparison to PS1KI^ho^ mice harboring the same PS1 knock-in mutation in the absence of human APP expression and Aβ accumulation (Figure 9C).

## Discussion

Axonal deficits and impairment of motor performance are common pathological alterations in mouse models expressing different isoforms of human mutant tau protein (reviewed in Wirths and Bayer, 2008). However, in recent years, similar phenotypes have been reported for AD mouse models based on APP overexpression (Stokin et al., 2005; Wirths et al., 2006, 2007; Adalbert et al., 2009; Jawhar et al., 2012) and disturbances of axonal transport rates have been reported in APP-based transgenic mouse models of Down syndrome (Salehi et al., 2003) and AD (Smith et al., 2007; Kim et al., 2011; Wang et al., 2012). Accordingly, APP has been demonstrated to undergo fast axonal transport (Koo et al., 1990), presumably by a kinesin-I-mediated mechanism (Kamal et al., 2000), and the β-site cleaving enzyme (BACE) and PS1 have been shown to be associated with APP-resident membranous cargos. This implies that Aβ can be produced directly within axons (Kamal et al., 2001), however, this finding has been questioned by others (Lazarov et al., 2005). In support of this hypothesis, APP, BACE, PS1, and caspase-3 were found to co-accumulate in swollen axons following traumatic brain injury (Chen et al., 2004).

It has been suggested that axonopathies in APP-transgenic mouse models entirely depend on APP overexpression with co-expression of FAD-linked PS1 mutants, leading to increased Aβ levels, thereby suppressing axonal defects (Stokin et al., 2008). This observation prompted us to analyze axonal degeneration in the APP/PS1KI^ho^ mouse model, as well as in APP single transgenic and APP/PS1KI^he^ mice, all expressing the same APP transgenic construct at equal levels. Previous work in the APP/PS1KI^ho^ mouse model has demonstrated a severe age-dependent axonal degeneration phenotype, which is characterized by the accumulation of large axonal swellings (Wirths et al., 2007). These swellings were most abundant in fiber-rich regions of the central nervous system such as corpus callosum, pons, medulla and spinal cord. We demonstrated a significant increase in these swellings between 6 and 10 months of age, which only marginally worsened at the age of 14 months (Wirths et al., 2007). The mice used in the present study are based on Thy1-driven overexpression of APP751 with the Swedish and London mutations and carry either no (APP), one (APP/PS1KI^he^), or two (APP/PS1KI^ho^) mutant murine PS1 alleles under the control of the endogenous PS1 promoter (PS1 “knock-in,” PS1KI). Using this strategy, we ensured that the APP expression levels did not differ between the mouse lines, which we confirmed by immunostaining and western-blotting using APP antibodies. However, the amount of Aβ peptides and APP C-terminal fragment levels differs significantly between the analyzed mouse lines, leading to dramatic increases in the Aβ load, comprising plaques as well as intraneuronal Aβ, as a function of mutant PS1KI gene dosage. It has been previously shown that expression of mutant PS1 alleles in combination with the APP transgene does not only lead to a higher overall Aβ load, but in addition causes a significant rise in the Aβ 42/Aβ 40-total ratio and an earlier plaque onset (6 m in APP vs. 2 m in APP/PS1KI^ho^ mice) (Casas et al., 2004).

To facilitate quantification of axonal defects we introduced APP phosphorylated at threonine 668 as a novel marker for axonal spheroids and demonstrated that the detected axonal swellings perfectly overlap with APP, a widely used marker for axonal degeneration that is employed in routine neuropathological analysis. In contrast to APP, which is abundantly present in the somato-dendritic compartment of neurons, pAPP has been predominantly detected in neuritic processes, including axonal spheroids, as well as mossy fibers. This corroborates previous studies, as pAPP has been previously shown to be present predominantly in neurites and growth cones of PC12 cells (Ando et al., 1999), as well as in dystrophic neurites decorating senile plaques in APP transgenic mice (Shin et al., 2007). Using double-immunofluorescent stainings with N- and C-terminal APP antibodies, a complete overlap was detected in axonal spheroids, making it impossible to draw conclusions about the sole presence of APP CTFs. The Aβ [N] antibody detects the N-terminus of Aβ (aspartate at position 1) and it might therefore be assumed that this antibody is also capable of detecting APP CTFs. However, we have previously demonstrated that no immunoreactivity could be detected in a mouse model overexpressing APP CTFs (SPA4CT mice), which strongly argues against a cross-reactivity, confirming that the Aβ [N] antibody detects only Aβ peptides using immunohistochemical methods (Christensen et al., 2010).

Upon quantification of pAPP, as well as NF-200-positive axonal swellings distant from plaques in the pons and spinal cord, significant increases in the numbers of dystrophic spheroids were detected in APP/PS1KI^he^ mice, compared to APP single transgenic mice which were almost devoid of any axonal swellings in the analyzed regions. Comparison of APP/PS1KI^he^ with APP/PS1KI^ho^ revealed a further 2–4 times increase of spheroids in both pons and spinal cord.

This result is in apparent contradiction to the published finding that the level of axonal defects was unchanged or even suppressed in mice expressing mutant PS1 in combination with mutant APP (Stokin et al., 2008). One major difference between the APP/PS1 double-transgenic mice used in this report and our mouse model is the fact that the endogenous murine PS1 gene was still present in the APP/PS1 transgenic mice, whereas it had been sequentially replaced by the FAD mutant form in the APP/PS1KI model used in the present study. On the other hand, APP/PS1KI^he^ mice still harbor one copy of endogenous PS1 but already show a dramatic aggravation of the axonal degeneration phenotype. It has been hypothesized that kinesin-based axonal transport is compromised by mutations in PS1 via interaction with glycogen synthase kinase 3β (GSK3β) and it has been further demonstrated that the relative levels of GSK3β activity were increased in the presence of mutant PS1, as well as in the absence of wildtype PS1, resulting in increased phosphorylation of kinesin-light chain and reduced anterograde transport (Pigino et al., 2003). In addition, anterograde fast axonal transport of APP and Trk receptors is impaired in the sciatic nerve of mice expressing FAD-linked PS1 mutations, resulting in an increased phosphorylation of tau and neurofilaments in the spinal cord (Lazarov et al., 2007). We were able to demonstrate KLC1 accumulations in APP/PS1KI^he^ and APP/PS1KI^ho^ mice, whereas PS1KI^ho^ or APP single transgenic mice were completely devoid of KLC1-positive axonal swellings. This implies that Aβ accumulation plays a critical role in the development of the axonal phenotype in contrast to the pure presence of mutant APP or mutant PS1. This is corroborated by the reduced KIF1A and KIF5A mRNA levels indicating axonal transport alterations in APP/PS1KI^ho^ mice.

In contrast to a major role for APP in the development of axonal degeneration, our data is more consistent with a major influence of Aβ peptides resulting in the abovementioned phenotype. Previous studies have linked Aβ peptides with axonal transport deficits (e.g., Vossel et al., 2010) and recently especially oligomeric Aβ peptides have been linked to impaired axonal transport. In good agreement with our finding of oligomeric intracellular Aβ in axonal spheroids and respective neuronal cell bodies, Pigino and colleagues have demonstrated that intraneuronal oligomeric Aβ, but not unagreggated or fibrilar Aβ species, compromises retrograde and anterograde fast axonal transport in isolated squid axoplasm in nanomolar concentrations. This has been mechanistically linked to activation of casein kinase 2 (CK2), which in turn leads to increased phosphorylation of kinesin-1 light chains (KLC1) and subsequent release of kinesin from its cargoes (Pigino et al., 2009). Moreno and co-workers demonstrated that nanomolar concentrations of intraaxonal oligomeric (o)Aβ 42, but not oAβ 40 or extracellular oAβ 42, acutely inhibited synaptic transmission (Moreno et al., 2009). In addition, recent studies implying primary hippocampal neurons demonstrated a marked decrease in axonal trafficking of dense-core vesicles and mitochondria in the presence of oligomeric Aβ (Decker et al., 2010). Furthermore, Aβ peptides were found to accumulate in axonal swellings distant from plaques, as well as in the vicinity of plaques, raising the possibility that these swellings precede and contribute to the formation of extracellular Aβ deposits. YFP-H mice express the fluorescent protein YFP under the control of the murine Thy1 promoter in a subset of cortical and hippocampal neurons (Feng et al., 2000) and greatly facilitated the tracking of neuronal processes from their respective cell bodies (Beirowski et al., 2004; Adalbert et al., 2009) in the present study. Aβ-positive granules were also detected in the axons and apical dendrites of cortical neurons, leading to the assumption that Aβ can be transported within fibers. It has been hypothesized that if Aβ generation occurs at the sites of axonal blockage, amyloid deposition might be due to focally increased Aβ secretion or lysis of axonal spheroids that were enriched in Aβ peptides (Stokin et al., 2005). Consistent with this hypothesis, Xiao and co-workers have demonstrated by post-mortem tracing in human AD brains, that tracer staining was observed around swollen axons, a type of axonopathy, referred to as “axonal leakage.” In these leaking axons, the tracer staining was observed in both intra-axonal and extra-axonal spaces and even in the myelin sheath, while it was restricted to the intra-axonal space in intact axons (Xiao et al., 2011). Our finding of Aβ accumulation in fibers together with diffuse extracellular Aβ deposits in the close vicinity of axonal spheroids might add further evidence to the hypothesis that Aβ can be released from fiber swellings, however more research is needed to substantiate such a mechanism. It has been demonstrated that Aβ-induced neuronal dysfunction can progress from the neurons in the entorhinal cortex that selectively overexpress amyloid precursor protein (APP) to the terminal end of the dentate gyrus in a trans-synaptic manner (Harris et al., 2010). In addition, Aβ reduces the number of spines and inhibits synaptic plasticity in neurons that neighbor neurons overexpressing APP (Wei et al., 2010). Finally, microinjections into electrophysiologically defined primary hippocampal rat neurons demonstrated the direct neuron-to-neuron transfer of soluble oligomeric Aβ followed by subsequent cytotoxicity (Nath et al., 2012). Taken together, there is ample evidence that intracellular and in particular intraxonal Aβ peptides might play in important role in the neurodegenerative processes underlying AD.

### Conflict of interest statement

The authors declare that the research was conducted in the absence of any commercial or financial relationships that could be construed as a potential conflict of interest.
